# The influence of four pharmaceuticals on *Chlorellapyrenoidosa* culture

**DOI:** 10.1038/s41598-018-36609-4

**Published:** 2019-02-07

**Authors:** Yonggang Zhang, Jun Guo, Tianming Yao, Yalei Zhang, Xuefei Zhou, Huaqiang Chu

**Affiliations:** 1 School of Chemical Science and Engineering, Tongji University, Shanghai, 200092 China; 2 State Key Laboratory of Pollution Control and Resource Reuse, Tongji University, Shanghai, 200092 China

## Abstract

There has been a developing technology in algae with pharmaceuticals wastewater. However, the effect and the underlying mechanism of pharmaceuticals on algae are not well understood. To investigate the effect and mechanism of pharmaceuticalson microalgae, four pharmaceuticals of clofibric acid (CLF), ciprofloxacin (CIP), diclofenac (DCF) and carbamazepine (CBZ) on *C. pyrenoidosa* culture were analyzed. At low concentrations (<10 mg/L), the pharmaceuticals, especially the DCF, exhibited positive effects on both the structure and function of algal cultures; algal growth (i.e., chlorophyll *a* accumulation, lipid accumulation) and activities of antioxidant enzymes were stimulated. The algal metabolite differences of various DCF concentrations were investigated and a total of 91 substances were identified, whose samples were clustered and clearly separated. The key metabolomics pathway analysis found that the DCF promoted the carbohydrate and fatty acid metabolic pathway in *C. pyrenoidosa* under relatively low concentrations (<10 mg/L). However, the algae metabolomics pathway was disturbed significantly under the action of a high concentration of DCF (>100 mg/L). The study detected the effects of four pharmaceuticals on *C. pyrenoidosa* and demonstrated that the usage of metabolomics analysis complemented with DCF could be an effective approach to understand the mechanism of molecular evolution in *C. pyrenoidosa* for microalgal biomass and bioenergy from wastewater in researches of biological resources.

## Introduction

The decline of fossil fuel reserves and increasing concerns about both future fuel security and the environmental impacts associated with greenhouse gas emissions have driven the development of alternative fuel technologies around the sustainable use of plant biomass^1,2^. As a biomass material and renewable carbon-neutral fuel, algae are a most promising feedstock for next generation biofuels because many algal species have higher biomass productivity than terrestrial crops, and algae can grow on marginal land and water bodies, resulting in less competition with food production for arable land^3^. However, there is still no consensus that commercial-scale production of algal biofuels is environmentally beneficial^4^. Recently, numerous studies have suggested using nutrient-laden wastewater in algae cultivation to improve the environmental performance of algal biofuels^5–8^ by re-using waste nitrogen (N), phosphorus (P), and water, reducing the energy use and emissions from acquiring these inputs, and potentially reducing overall biofuel production costs. Various wastewaters, such as municipal and industrial wastewater, have been examined for algal cultivation^4,9^. Due to their compositional complexity, these wastewaters increase the difficulty and uncertainty of algal biomass cultivation^4^.

Our previous studies have shown that *Chlorellapyrenoidosa* (*C. pyrenoidosa*) is well-adapted to anaerobic digested starch processing wastewater and is the dominant microorganism in the photobioreactor in outdoor batch and continuous cultures. Zhang *et.al*. found that CBZ from 1, 2, 5 and 10 mg/L significantly inhibited the growth of *C. pyrenoidos*^10^. A study from Lai reported that chloramphenicol inhibited the growth of *C. pyrenoidosa* at concentrations of 20 and 40 mg/L and did not affect the growth of *C. pyrenoidosa* at concentrations of 2.5, 5, and 10 mg/L^11^. The lipid contents of the harvested biomass can range between20% to 25% of their dried weight^12,13^. The productivity of the targeted bioproducts from microalgae should be improved to make this process economically feasible and sustainable. Early studies have aimed to identify and apply chemical triggers or enhancers to improve cell growth and accumulation of bioproducts in microalgae. These studies demonstrate that the application of chemical triggers or enhancers can be a practical method for large-scale cultivation of microalgae^14^.

In this study, the interaction between pharmaceutical products and microalgae was studied. Four kinds of typical pharmaceutical products, clofibricacid (CLF), ciprofloxacin (CIP), diclofenac (DCF) and carbamazepine (CBZ), and one kind of common microalgae *C. pyrenoidosa*, were chosen for the experiment. The effect of these pharmaceutical products on *C. pyrenoidosa*, including the biomass, particle size distribution (PSD), accumulation of chlorophyll *a*, and lipid content, were studied. In addition, the metabolic mechanisms related to *C. pyrenoidosa* cell growth production and accumulation of bioproducts was assessed.

## Results and Discussion

### Effects of four PPCPs on PSD of *C. pyrenoidosa*

**Figure 1:**
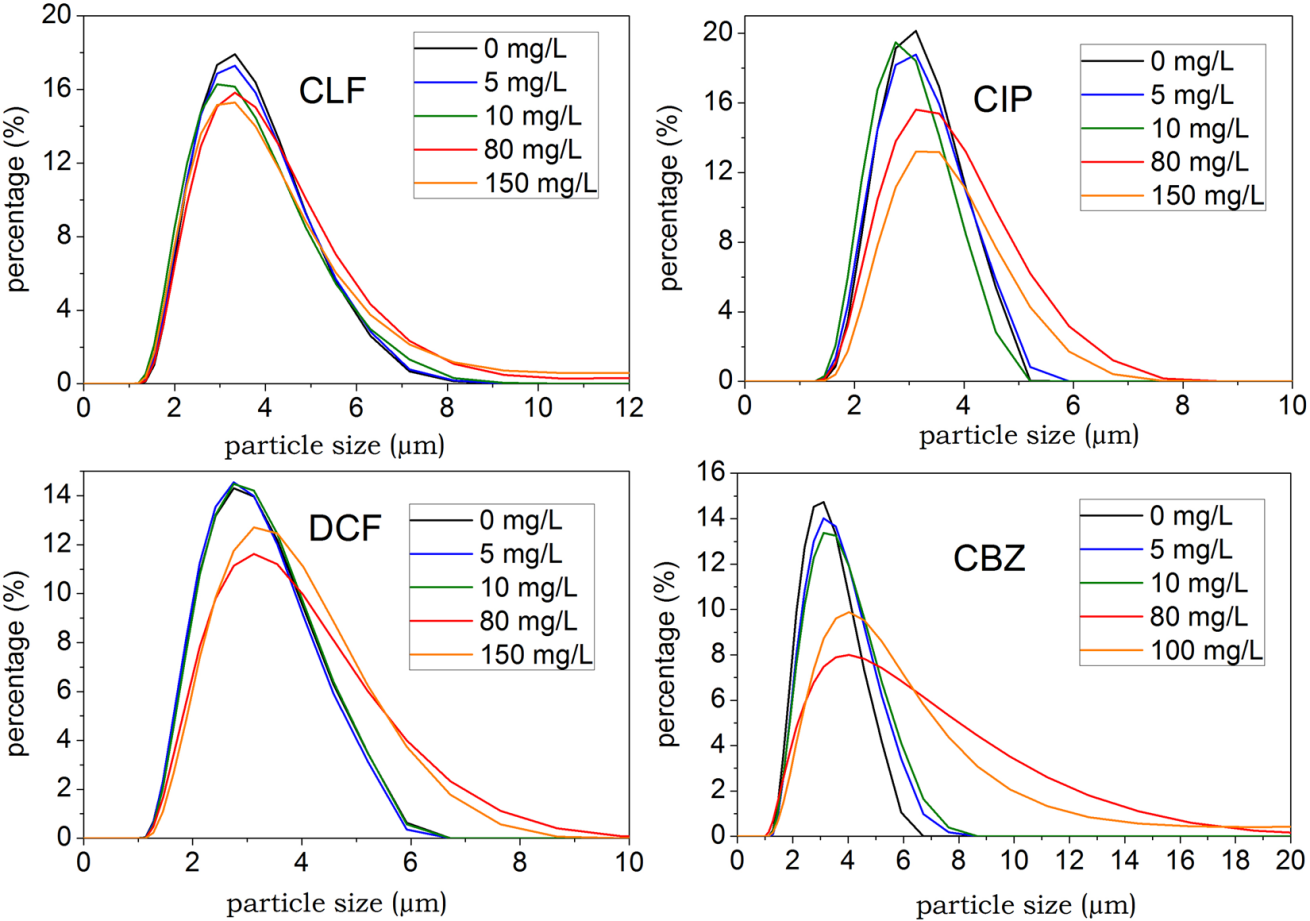
Particle size distributions of algal cultures with different concentrations of PPCPs.

The PSD, including unicellular cell size and colony size, of algal culture is an important indicator of algae growth conditions. The algal particle size significantly influences the adsorption behavior to toxicants^15^. Conversely, the effects of toxicants to algae cells can be reflected by PSD. Figure 1 shows the PSD of algal cultures of four pharmaceuticals. For the control group, the PSD range concentrated at 2.5–5.5 μm, and the proportion of *C. pyrenoidosa* individuals with a particle size greater than 10 μm tends to zero, which represents the normal cell size of *C. pyrenoidosa*. For drug dosing groups, PSD ranges greater than 10 μm gradually appeared, and with increasing PPCP concentration, the proportion of PSD greater than 10 μm increased at different degrees. In addition to the CIP test groups, the other three PPCP test groups of 80 mg/L and 150 mg/L have specific proportions of *C. pyrenoidosa* with a particle size of more than 10 μm, and the peaks of the new particle size distribution are at 15 μm and 40 μm. These drugs present several factors that affect algae aggregation. High concentrations of toxicants can damage the integrity of cell walls, lead to cell aggregation, or cause cell division dysfunction^16^. The excretion of algogenic organic matters, both the extracellular and intracellular organic matters, can increase under adverse conditions and cause algae aggregation, mainly four-celled and eight-celled colonies^17,18^. According to observations of algal culture morphology, treatment with a high drug dosage concentration would enhance algal coagulation and sedimentation, severely obstructing algae growth. In addition, cell size expands, as shown in Fig. 1. The phenomenon of cell volume enlargement coincides with results found in other studies that all show that algae swelling is a dominant effect of toxicants on microalgae^16,19,20^.

### Effects of four PPCPs on the growth, chlorophyll *a* and lipid of *C. pyrenoidosa*

**Figure 2:**
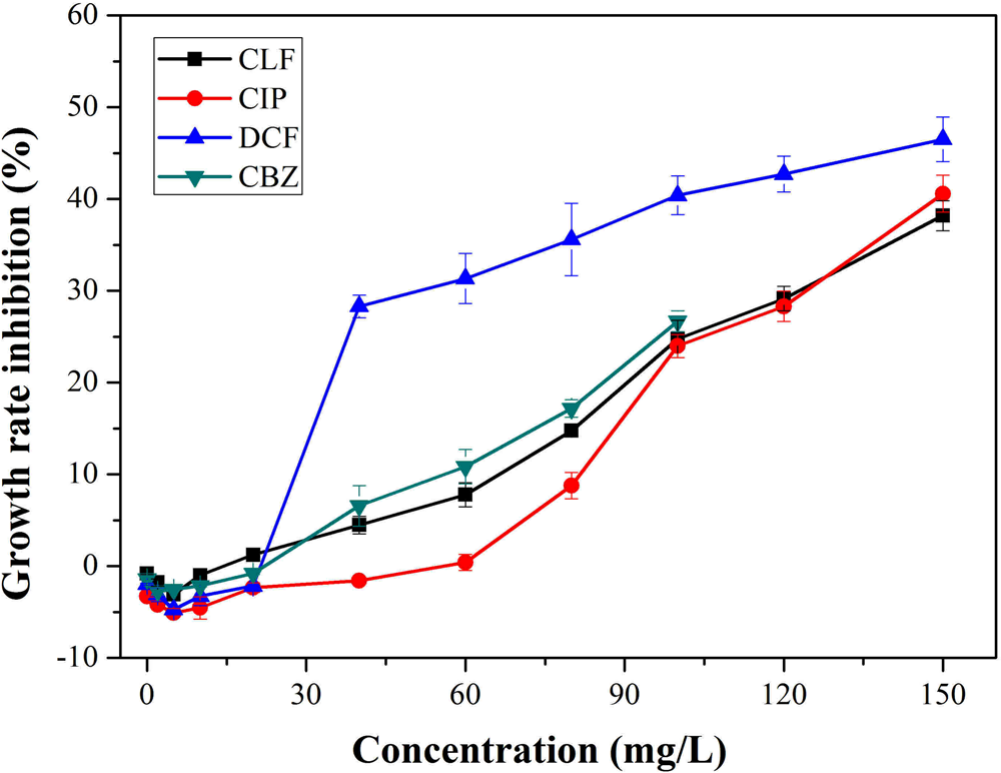
Growth rate inhibitions of various pharmaceutical concentrations in algal cultures. Error bars represent standard deviation (n = 3).

The biomass concentrations of *C. pyrenoidosa* with different contents of four PPCPs were compared with control group (Table 1). After 8 days culture, all treatments showed similar growth trend. Promotion on algae growth was observed in culture of low dose of four PPCPs and the highest biomass treated by CLF, CIP, DCF and CBZ was 2.11 g/L, 2.21 g/L, 2.23 g/L and 2.08 g/L with concentrations of 10 mg/L, 10 mg/L, 5 mg/L and 5 mg/L. Increasing concentrations of CLF, CIP, DCF and CBZ was confirmed by a reduction in biomass. Significant reduction of algae growth was observed when the contents of CLF, CIP CBZ were higher than 100 mg/L, while the biomass concentration of DCF significantly decreased under contents higher than 40 mg/L. As showed in Fig. 2, exposure to low concentrations of four PPCPs (2–30 mg/L) promoted the growth of *C*. *pyrenoidosa*. Furthermore, CLF, CIP, DCF and CBZ inhibition of cells growth increased with increasing compound concentrations over than 40 mg/L. However, the growth rate inhibition of DCF increased more significantly than CLF, CIP and CBZ. These results indicated that exposure to low doses of PPCPs could promote the growth of *C. pyrenoidosa*, suggesting an inductive effect of pharmaceutically active compounds on cells named as hormesis^21^ and high doses of PPCPs may cause the death of cells.

**Table 1:** Biomass concentration (g/L) of *C. pyrenoidosa* with various pharmaceutical concentrations after 8 days in algal cultures (*p < 0.05. **p < 0.01). Error values represent standard deviation (n = 3).

Concentrations (mg/L)	CLF	CIP	DCF	CBZ
0	1.96 ± 0.21	1.96 ± 0.21	1.96 ± 0.21	1.96 ± 0.21
2	1.98 ± 0.14	2.11 ± 0.15	2.04 ± 0.18	2.01 ± 0.07
5	2.03 ± 0.08	2.16 ± 0.11	2.23 ± 0.08	2.08 ± 0.12
10	2.11 ± 0.23	2.21 ± 0.22	2.19 ± 0.14	2.07 ± 0.09
20	1.99 ± 0.07	2.18 ± 0.17	2.11 ± 0.22	2.05 ± 0.14
30	1.88 ± 0.13	2.06 ± 0.11	2.05 ± 0.05	1.98 ± 0.21
40	1.73 ± 0.14	2.02 ± 0.08	0.94 ± 0.08*	1.64 ± 0.12
60	1.59 ± 0.21	1.92 ± 0.03	0.87 ± 0.03*	1.47 ± 0.02
80	1.33 ± 0.07	1.55 ± 0.15	0.78 ± 0.01**	1.25 ± 0.01
100	1.03 ± 0.05*	1.05 ± 0.11*	0.69 ± 0.01**	0.98 ± 0.05*
120	0.92 ± 0.07*	0.94 ± 0.08*	0.65 ± 0.02**	/
150	0.73 ± 0.13**	0.62 ± 0.01**	0.59 ± 0.06**	/

**Figure 3:**
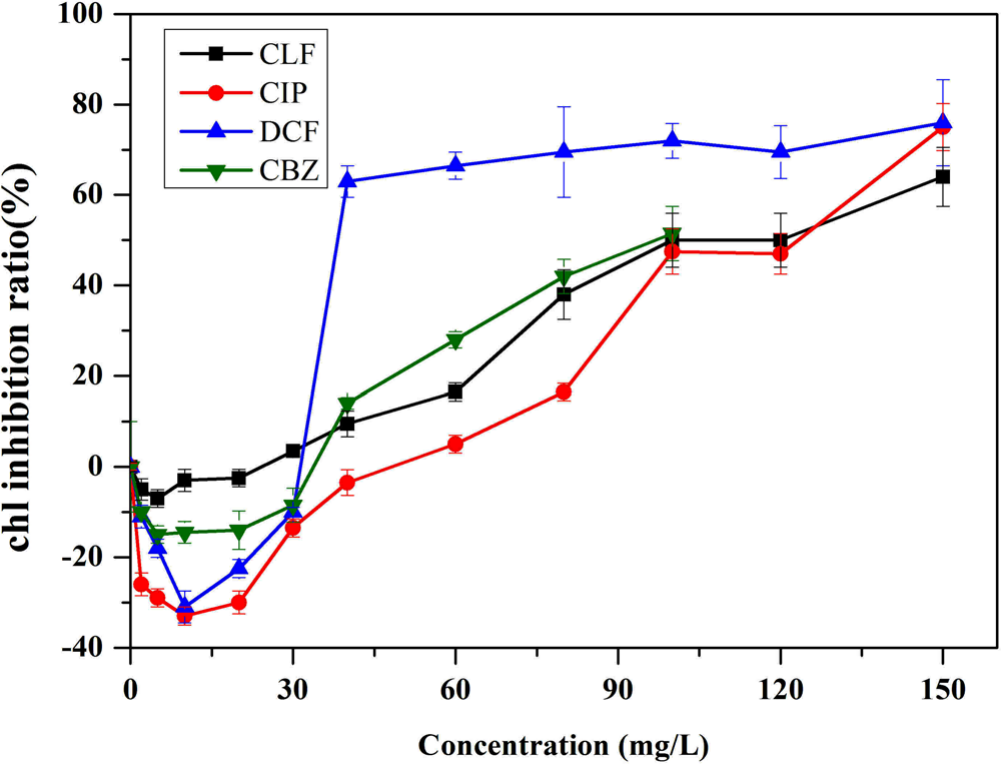
Chlorophyll *a* inhibition rates of various pharmaceutical concentrations in algal cultures. Error bars represent standard deviation (n = 3).

For photosynthetic microalgae, chlorophyll *a* is the primary photochemically active compound, and it behaves as a receiver of light for driving photosynthesis. The content of chlorophyll *a* influences the production of biomass and the accumulation of target compounds^22^. In previous studies it was elucidated that chlorophyll *a* is the primary target for growth rate and toxicity, resulting in a decrease in photosynthesis and reduced growth^23^. Figure 3 shows the chlorophyll *a* inhibition rates of various pharmaceutical concentrations to algal cultures. At low concentrations, CLF,CBZ (<10 mg/L) and DCF and CIP(<30 mg/L) play a positive role in chlorophyll *a* accumulation and stimulate algae growth. The highest stimulation rates of DCF and CIP were 30.3% and 31.7%, respectively. The stimulating effects of compounds on green algae have been found in other studies. The growth of *C. reinhardtii* was stimulated at low levels of fluroxypyr (0.05 to 0.5 mg/L)^24^. At concentrations as low as 0.10 mg/L, diclofop-methyl significantly stimulated algal growth by up to 40%^25^. Another study showed that the growth rates of *C. pyrenoidosa* in CBZ treatment were slightly increased at 96 h^10^. This phenomenon can be explained by other findings. One study found that some compounds provide the carbon/phosphorus sources for the algae culture^26^. Another study showed that some compounds might enhance enzyme activities and thus, increase the synthesis of DNA, RNA and proteins^25^. Compared to DCF and CIP, CLF did not have an obvious stimulating effect on *chlorophyll a* concentration. Chlorophyll *a* inhibition rates of CLF, DCF and CBZ obviously increased under concentrations higher than 30 mg/L, while chlorophyll *a* inhibition rate of CIP significantly increased under concentrations higher than 40 mg/L. The inhibition rates of DCF at concentrations of 40 mg/L reached approximately 70%, while the inhibition rate of CIP at 100 mg/L was only 47.0%, demonstrating that CIP had relatively low toxicity to the synthesis of chlorophyll *a* and growth of algal cultures at high concentrations. Compared to the change of algae concentration, a good concomitancy was noted between algae growth and chlorophyll *a* content in present experiment. It was considered that the inhibition of algal growth resulted from the reduction of chlorophyll biosynthesis and subsequent peroxide destruction of thylakoid membranes when induced by toxic compounds^27–29^.

Algal lipid concentrations ranged from 5% to 70% of dry weight and were mostly commonly between 15% and 30%^30^. As a potential energy alternative to the diminishing world fossil fuel reserves, microalgae have been investigated as a source of biodiesel. Green algae, especially *Chlorella* and *Chlamydomonas*, have been shown to be ideal sources of lipids suitable for biodiesel production^31^. Lipid content of algal cells can be affected by growth phase, nutrients, and other physical and chemical factors^32^.

**Figure 4:**
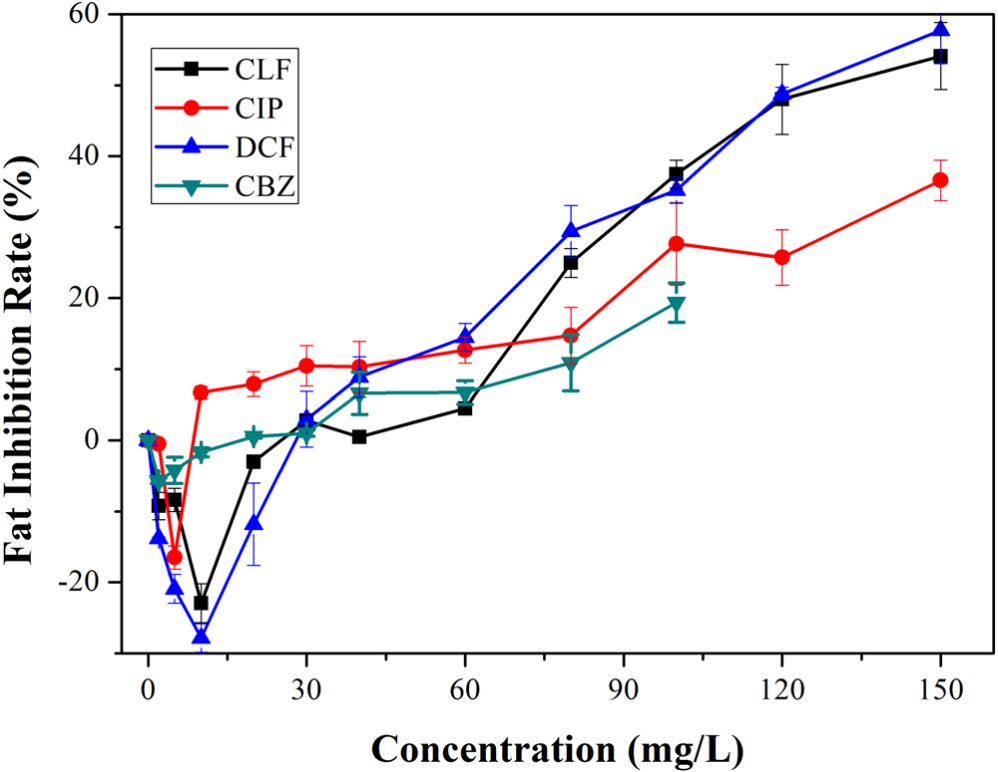
Lipid yield inhibition rates of various pharmaceutical concentrations in algal cultures. Error bars represent standard deviation (n = 3).

Figure 4 shows the total lipid yield inhibition rates of various pharmaceutical dosages. At concentration lower than 30 mg/L, CLF, DCF and CBZ positively affected lipid accumulation in algal cells, and CIP had a stimulating effect on lipid accumulation that only occurred when the concentration was less than 10 mg/L. The trend for the stimulating rates of lipid yield was DCF > CLF > CBZ > CIP, and the highest rate was 27.9%. Stimulation could be observed at a wide concentration range, from 2 mg/L to 30 mg/L. This could be due to the pharmaceuticals or their metabolites that served as sources of nitrates or phosphates to enhance lipid productivity^33^ or due to changes in relevant enzyme activities that led to a buildup of lipid compounds. The detailed mechanisms still remain unclear. Some fungicides, industrial chemicals and types of radiation have also been found to have stimulating effects on algae lipid accumulation^16,19^. All groups of CLF, CIP, DCF and CBZ inhibited the production of lipid at concentrations higher than 30 mg/L, but the lipid content of DCF changed more obviously within 30 mg/L. The former effect may be due to the high concentration of pharmaceuticals partially damaging the integrity of algae cells and blocking lipid synthesis^30^.

### Effects of for PPCPs on SOD and CAT activities

Various physical and chemical stress, such as metals, radiation and organic compounds, have been found to influence the growth of microalgae. These stresses could enhance the production of reactive oxygen species (ROS), including superoxide radical (O_2_^−^), hydrogen peroxide (H_2_O_2_) and hydroxyl radical (·OH)^34,35^. To regulate and protect against the damaging effects of ROS, a suite of antioxidant enzymes and substances exist in living cells. The SOD-CAT system serves as the first line of defense against oxygen toxicity and is an indicator of ROS production^36^. SOD, which performs the first step in removing ROS, converts O_2_^−^ to H_2_O_2_ and oxygen. CAT degrades H_2_O_2_ to oxygen and water. There have been several studies reporting the effects of organic pollutants on antioxidant enzymes in green algae^10,24,34^.

**Figure 5:**
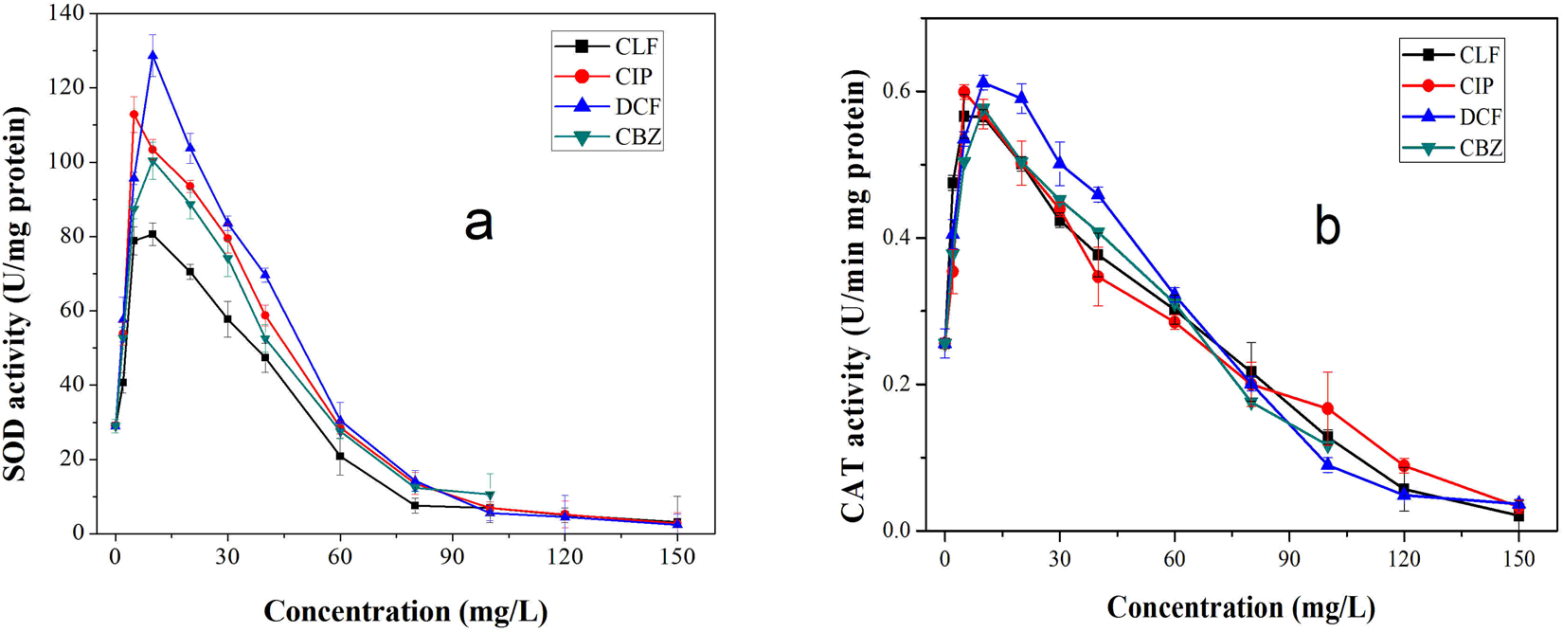
(**a**) SOD and (**b**) CAT activities from exposure to different concentrations of four pharmaceuticals. Error bars represent standard deviation (n = 3).

Figure 5 shows the exposure of SOD and CAT activities to different contents of four pharmaceuticals. The SOD activities of algae cultures peaked at a dosage of 10 mg/L for all pharmaceuticals. With dosages higher than 40 mg/L, the SOD activities dropped sharply. At dosages of 60 mg/L, the content of SOD was lower than that of the control group. CAT activities showed similar trends with SOD. The activities of the enzymes coincided with algae growth inhibitions. The increased SOD and CAT activities were due to the defense mechanism responding to increasing concentrations of ROS, suggesting that the cellular tolerance to oxidative stress was initiated to cope with excess production of O_2_^−^ and H_2_O_2_. However, under high concentrations, severe contact damage appeared, due to either experiencing extreme intensities of stress or having reached the stage of exhaustion, and all contributed to the sharp decrease of SOD and CAT activities^34^.

### Metabolomic analysis of *C. Pyrenoidosa* under DCF addition

**Figure 6:**
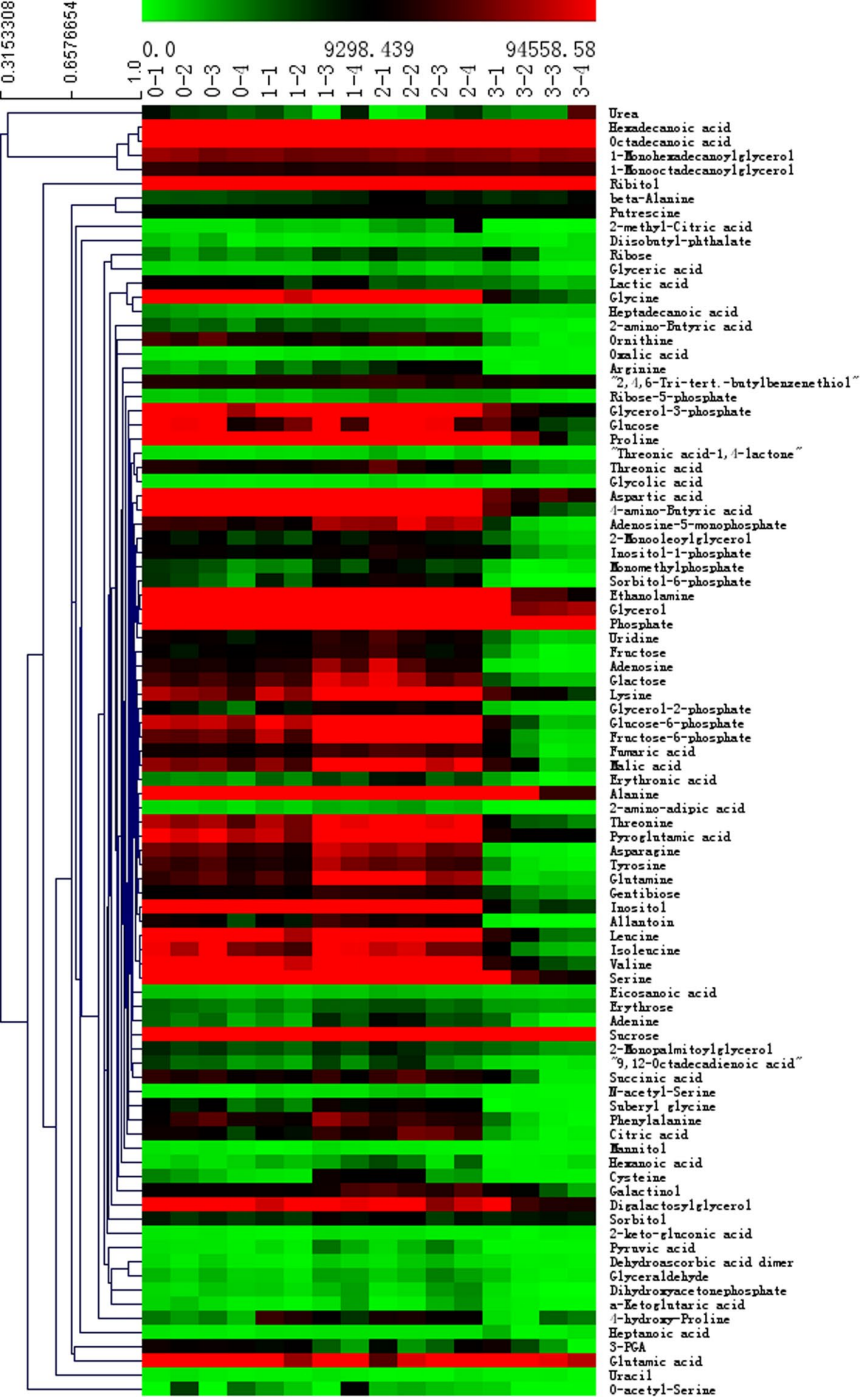
The heat map of the four groups of metabolites. The concentrations of DCF for group 0 to 3 was 0 mg/L, 5 mg/L, 10 mg/L, and 100 mg/L, respectively. The color bar legend means Row Z-Score. All of groups were repeated four times.

Analysis of biomarkers. From the aforementioned results, the cells under DCF condition showed obviously changes in algae growth. Metabolic response could effectively reveal the molecular mechanism of *C*. *pyrenoidosa* induced by DCF. In this study, GC-MS was used to identify the algal metabolite differences of various DCF concentrations for 8 days. After preprocessing, a total of 91 substances were identified that are mainly endogenous metabolites, including organic acids, alcohols, sugars, fatty acids, and amino acids. Based on the metabolites measured by GC-MS, the metabolic heat maps of the 16 samples at different DCF concentrations are shown in Fig. 6. We found evident differences in the composition and content of metabolites. Among them, most of the metabolites had an important relationship to the algal carbohydrate metabolism, amino acid metabolism, lipid metabolism, nucleotide metabolism and other major metabolic pathways. Compared with the control group, the content of most metabolites increased at a DCF dose of less than 10 mg/L. However, when the concentration was 100 mg/L, the content of most metabolites significantly decreased. Statistical methods are needed to determine if these metabolites are intrinsically related to the growth of *C. pyrenoidosa*.

**Figure 7:**
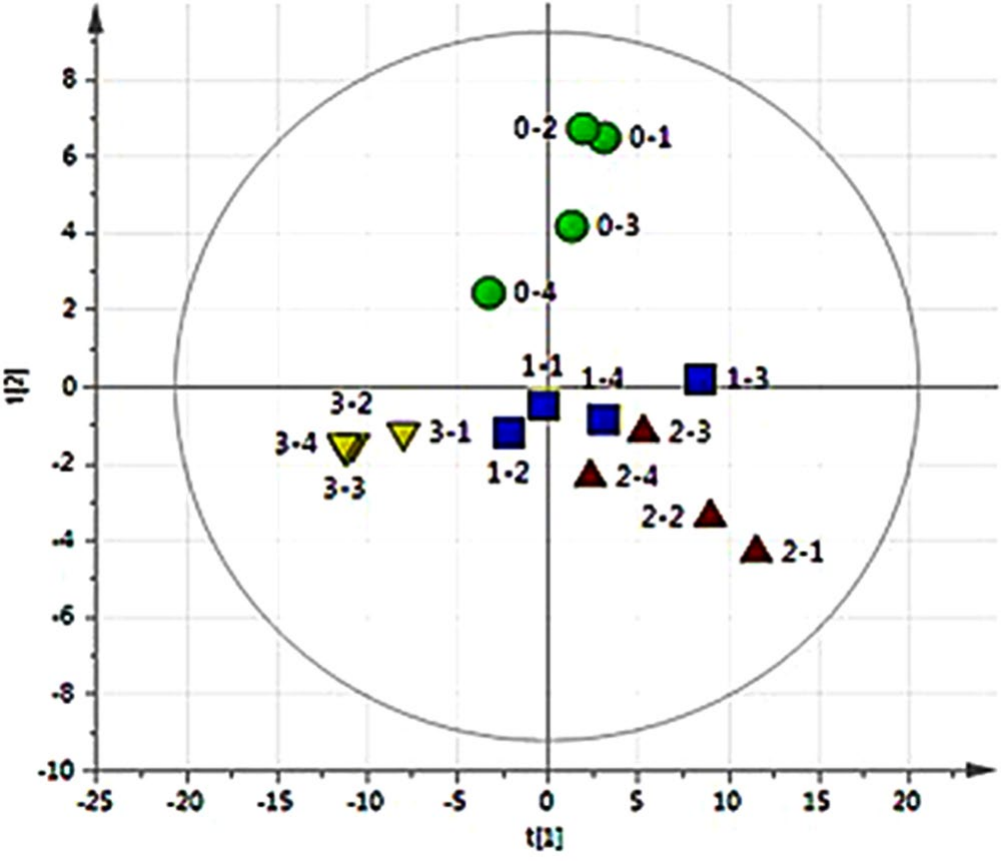
Four sets of samples depicted on a PCA (UA) model scatter plot. (R^2^X (cum) = 70.5%, Q^2^ (cum) = 57.6%). In the score plots, circularity represents 0 mg/L, box represents 5 mg/L, triangle represents 10 mg/L and inverted triangle represents 100 mg/L. The concentrations of DCF for group 0 to 3 was 0 mg/L, 5 mg/L, 10 mg/L, and 100 mg/L, respectively. All of groups were repeated four times. (*p* < 0.05).

Statistical analysis of the metabolites. The data were dimensioned and analyzed by SIMCA software, as shown in Fig. 7. The figure shows that in the 95% confidence interval, the four groups of samples were clustered and separated. The sample points of the same group were aggregated, and no abnormal points appeared. This demonstrated that the metabolites in four groups are indeed different. Sample points between group 0 and 3 are greatly separated, while group 1 and 2 showed closely differences. This indicated that the metabolite differences in *C. pyrenoidosa* gradually increased after 8 days of growth with increasing DCF concentrations.

PCA and PLS-DA models were used to analyze the metabolic differences between three different DCF concentrations (5 mg/L, 10 mg/L and 100 mg/L) and the blank control group. R^2^X represents the ability to interpret the model variable X; R^2^Y represents the ability to interpret the model variable Y; and Q^2^ (cum) represents the predictability of the cumulative model. A larger R^2^X is correlated to a smaller Q^2^ value. A small difference between the explained and predicted data indicated better model quality.

Figures S5–7 shows the PLS-DA scatter plot of groups 1, 2, 3 compared with group 0, respectively. No over-fitting occurred, indicating that the PLS-DA model is robust. To determine the biomarkers, the PLS-DA model was used to analyze the differences between the two groups with/without DCF, as shown in Figs S8–10. In the loading chart (Fig. S11), the points farther away from the origin indicated a greater difference between the two tested groups.

According to the VIP value, the numbers of different biomarkers (VIP value >1 was the biomarker) in groups 1, 2, 3 were 14, 17 and 12, respectively (Tables 5–7. In addition, alanine, aspartic acid and lysine contents in the *C. pyrenoidosa* cells were significantly increased, while the contents of glycine, serine and glutamic acid were significantly decreased, indicating that DCF causes interference to amino acid metabolism on the algal cells. In addition, the content of some carbohydratesin *C. pyrenoidosa*, such as sucrose, muscle sugar and 6-phosphate glucose, increased significantly, indicating that the DCF dose of 5 mg/L clearly affected the action of carbohydrate metabolism.

The effect of DCF dose of 10 mg/L on the metabolites of *C. pyrenoidosa* is similar to that of 5 mg/L, but the effect on the formation of certain carbohydrates and amino acids is more significant. The contents of phosphate, alanine, aspartic acid, ethanolamine, lysine, glucose-6-phosphate, phosphate and other substances increased significantly.

When the concentration of DCF in the culture medium was 100 mg/L, most metabolites in *C. pyrenoidosa* were significantly lower than those in the blank control group, demonstrating that high dose of DCF resulted in significant effect on metabolic activity of *C. pyrenoidosa*.

Analysis of the key metabolomics pathway. Further analysis of the metabolites was conducted with Metabo Analyst software, which is based on pathway enrichment analysis and pathway topology analysis in Fig. S12. The metabolic pathway of the 5 mg/L, 10 mg/L, 100 mg/L and blank control groups were shown in Tables. 8–10. A higher impact-value signifies a more relevant metabolic pathway. When the impact-value is set to 0.15, the main affected metabolic pathways of *C. pyrenoidosa* are screened out in the three groups.

**Figure 8:**
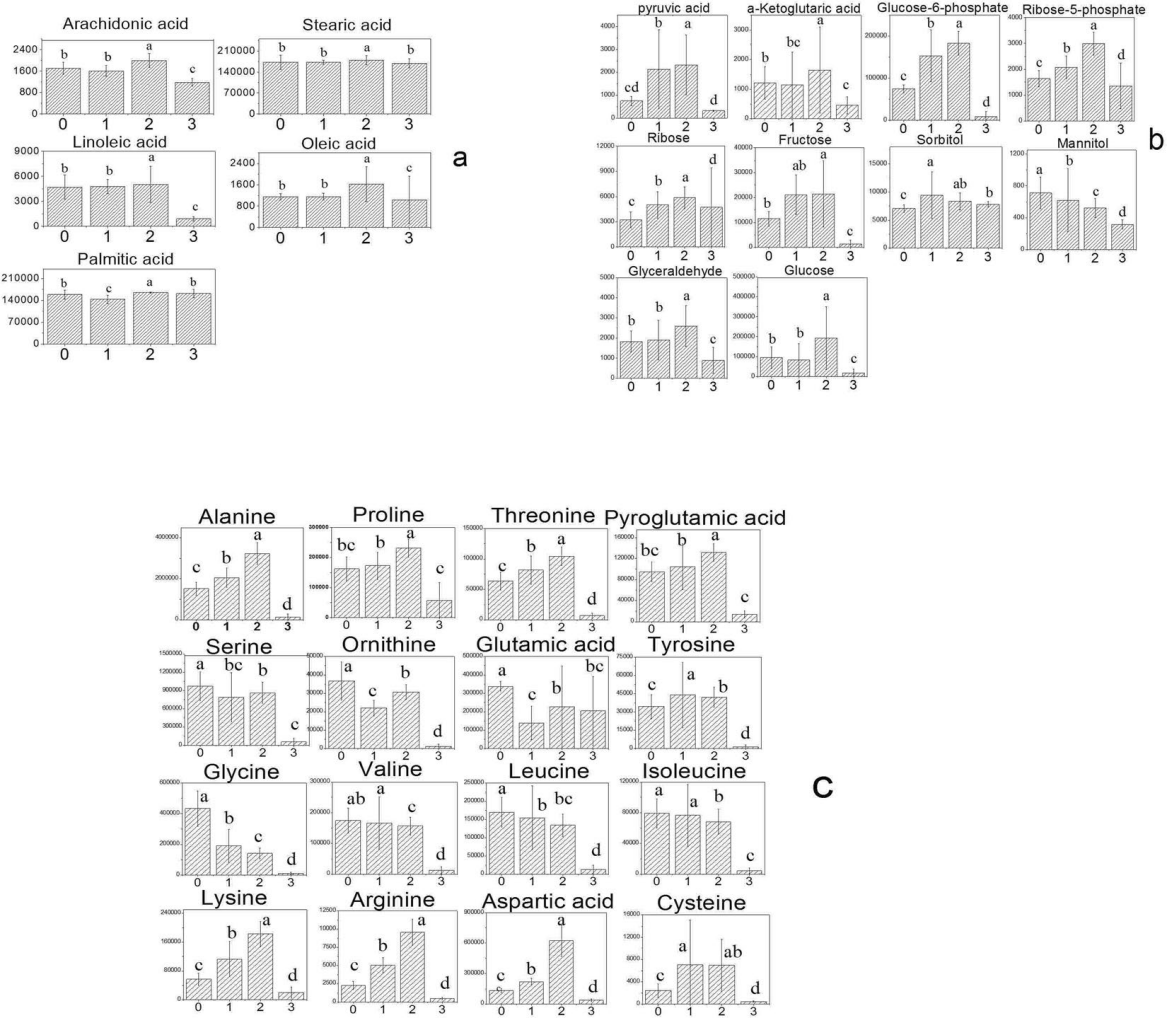
Changes in the main metabolites in fatty acid synthesis (**a**), TCA (**b**) and amino acid (**c**) processes under different DCF concentrations. The concentrations of DCF for group 0 to 3 was 0 mg/L, 5 mg/L, 10 mg/L, and 100 mg/L, respectively. The values annotated with different letters significantly difference (p < 0.05). Error bars represent standard deviation (n = 3).

In algae cells, the main sites of the fatty acid synthesis reaction are located in the plastid, in which the fatty acids used in the synthesis of fats and oils mainly include stearic acid, oleic acid, and linoleic acid. Subsequently, the synthetic free fatty acids are transported to the cytoplasm and then into the endoplasmic reticulum and other parts of the TAG synthesis. The results of the metabolic pathways associated with fat metabolism analysis were shown in Fig. 8a, which indicated that the content of fatty acid in cells was almost same as that in the blank control group under the effect of a DCF dose of 10 mg/L. At 10 mg/L DCF, the contents of fatty acids in *C. pyrenoidosa* were slightly higher than that in the blank control group. Under the action of 100 mg/L DCF, the contents of fatty acids, such as arachidonic acid and linoleic acid, were significantly decreased, indicating that the fatty acid metabolic pathway was disturbed induced by a high concentration of DCF. Metabolites in pyruvate metabolism and the tricarboxylic acid cycle (TCA), including pyruvate, malic acid, citric acid, fumaric acid, succinic acid, and α-ketoglutaric acid, firstly increased and then declined with the increasing concentration of DCF (Fig. 8b). It indicated that the pathway of carbohydrate was promoted in *C. pyrenoidosa* under low concentrations of DCF, and the normal physiological activities of *C. pyrenoidosa* were hindered with a higher concentration of DCF.

The amino acid metabolism-related metabolic pathways, such as the aminoacyl-tRNA biosynthetic metabolic pathways, glycine, serine and threonine metabolic pathway, lysine degradation, D-glutamine and D-glutamate metabolic pathways, valine, leucine and isoleucine degradation, are significantly affected by concentrations of 5 mg/L, 10 mg/L and 100 mg/L DCF (Fig. 8c). In addition, the porphyrin and chlorophyll metabolism pathway is significantly affected.

## Conclusions

Four kinds of PPCPs can promote the accumulation of oil and lipids in *C. pyrenoidosa* at a low concentration, and the effect of DCF on the accumulation of oil and lipids is the most significant and broad. In addition, the change of ROS at different concentrations of DCF reflects that algae cells can adapt the stimulation at a low concentration of PPCPs and the increase of SOD and CAT presents the antioxidant defense from microalgae induced by a high concentration of PPCPs. Under different concentrations of DCF, the metabolic pathway related to carbohydrate metabolism and amino acid metabolism is significantly affected.

## Materials and Methods

### Materials

The physical and chemical properties of the four test pharmaceutical products (CLF, CIP, DCF and CBZ) are shown in Table S1. *C*. *pyrenoidosa* (green algae, Collection No. FACHB-9) was obtained from the Institute of Hydrobiology at the Chinese Academy of Sciences (China). The cultivation process of *C*. *pyrenoidosa* is described in the Supporting Information.

### Test of operation factors for microalgae culture

To control for possible influencing factors in the experiment, the first test identified the factors that could affect the results, such as whether the SE medium, high-temperature sterilization, ultraviolet light or constant-temperature culture caused degradation of PPCPs (see Supporting Information Figs S1–S4. The results showed that SE culture medium, high-temperature sterilization and constant temperature during the culture process did not cause any loss of the four kinds of PPCPs or affect their detection. UV light degraded less than 4% of specific concentrations of CLF and CBZ. Second, according to the growth curve of *C. pyrenoidosa*, the growth rate of *C. pyrenoidosa* was stable until 8 days after inoculation, when the growth rate slowed significantly. Therefore, the culture period was set to 8 days.

### PPCPs influence test

First, the algae cells in the logarithmic growth phase were inoculated into 200 mL of the culture medium, so that the concentration was 0.15 g/L. Test solutions were obtained by separately spiking CLF, CIP, and DCF into the SE medium to achieve final concentrations of 0, 2, 5, 10, 20, 30, 40, 60, 80, 100, 120, and 150 mg/L for each of the three pharmaceutical products; CBZ was added to the SE medium to achieve final concentrations of 0, 2, 5, 10, 20, 30, 40, 60, 80, and 100 mg/L. The tests were replicated three times. Algal growth was determined by measuring optical density at 680 nm (OD_680_). After 8 day incubation, the algae samples were separated to determine biomass, growth rate, *chlorophyll a* concentration, lipid content, particle size distribution, SOD and CAT enzyme activity, elemental analysis and extracellular secretions to assess the metabolic effects of the four drugs on the *C*. *pyrenoidosa* culture.

The algal cells in the logarithmic growth phase were inoculated into the 200 mL test medium so that the algae concentrations reached 0.15 g/L, and the different concentrations of drugs (as described previously) were added. The test was repeated three times. After 8 d of incubation, 2 mL of the sample was removed from each test group and centrifuged at 5000 rpm for 10 min. The supernatant was filtered through a 0.22 μm membrane filter to remove residual cells, and then quantitatively analyzed by high-performance liquid chromatography. The concentration of each group of drugs was analyzed by liquid chromatography.

### Metabolomics analysis of DCF on *C*. *pyrenoidosa* cultures

DCF was dissolved in the SE medium to generate a concentration gradient of 0, 5, 10, and 100 mg/L and named as 0, 1, 2, and 3 groups, respectively. These were then autoclaved at 120 °C for 30 min and then sterilized by UV for 30 min. After inoculation of *C. pyrenoidosa*, the samples were cultured for 8 days, and the biomass concentration and chlorophyll *a* content were analyzed. The algae solution of each test group was centrifuged at 5000 r/min for 10 min, and the algae cells were washed three times with deionized water. After cryopreservation with liquid nitrogen, four groups of test samples were obtained. The subsequent detection methods, data processing methods and statistical analyses are described in the Supporting Information.

### Other analyses

In this study, the biomass concentration, chlorophyll *a* content, lipid content, SOD enzyme activity, CAT enzyme activity, particle size distribution, EOM, nitrogen content, and four concentrations of PPCPs were measured, and these are described in the Supporting Information.

## Electronic supplementary material

Supporting Information
