# Supplementary material for: The influence of four pharmaceuticals on Chlorellapyrenoidosa culture
Source: Sci Rep. 2019 Feb 7;9:1624. doi: 10.1038/s41598-018-36609-4 (PMC6367373; doi:10.1038/s41598-018-36609-4)
Supplement: Supplementary file 1 — Supporting Information [file 41598_2018_36609_MOESM1_ESM.doc]

Supporting Information

The influence of four pharmaceuticals on Chlorella pyrenoidosa culture

Yonggang Zhang^1^, JunGuo^2^, Tianming Yao^1^*, Yalei Zhang^2^*, Xuefei Zhou^2^, Huaqiang Chu^2^,

*^1^School of Chemical Science and Engineering,Tongji University,*

*Shanghai 200092, China.*

*^2^ State Key Laboratory of Pollution Control and Resource Reuse, Tongji University,* *Shanghai 200092, China.*

*Corresponding author phone: +86-21-65983292; fax: +86-21-65985811;

E-mail: tmyao@tongji.edu.cn (T. Yao); zhangyalei@tongji.edu.cn (Y. Zhang)

MATERIALS AND METHODS

### **Test PPCPs.** Information on the physical and chemical properties of the four PPCPs (CLF, CIP, DCF and CBZ) used in this study is shown in Table S1.

**Table S1** Physical and chemical properties of the four PPCPs.

|  | CLF | DCF | CIP | CBZ |
| --- | --- | --- | --- | --- |
| CAS number | 882-09-7 | 15307-79-6 | 85721-33-1 | 298-46-4 |
| Structural formula | 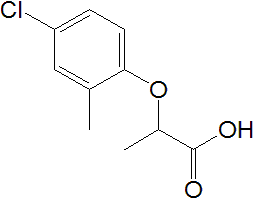 | 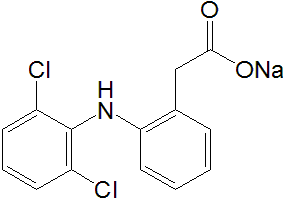 | 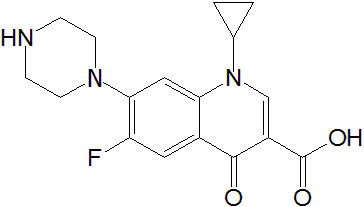 | 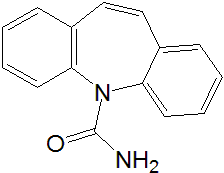 |
| Molecular weight | 214.65 | 318.14 | 331.35 | 236.28 |
| Solubility（mg/L） | 582.5 | 2425 | 11480 | 112 |
| pK_a_ | 2.84 | 4 | pK_a1_=6.1,pK_a2_=7.1 | 7 |
| log K_ow_ | 2.57 | 4.51 | 0.28 | 2.45 |

**Cultivation of *C. pyrenoidosa.*** *C. pyrenoidosa* was cultured in an SE medium, as shown in Table S2. Approximately 200 mL of green algae cultures were cultured in a 250 mL glass tank in an incubator at 30 °C，with light/dark of 14 h/10 h and a light intensity of 60μmol·m^-2^ s^-1^. To maintain the suspension of *Chlorella sorokiniana*/*pyrenoidosa* in the cultures, the bottle was shaken three times per day. The test algae culture was the SE medium. The medium formula is shown in Table S2.

**Table S2** Formula of the SE medium.

| Medicine | Concentration | Comment | |
| --- | --- | --- | --- |
| NaNO_3_ | 250 mg/L |  |  |
| K_2_HPO_4_·3H_2_O | 75 mg/L |  |  |
| MgSO_4_·7H_2_O | 75 mg/L |  |  |
| CaCl_2_·2H_2_O | 25 mg/L |  |  |
| KH_2_PO_4_ | 175 mg/L |  |  |
| NaCl | 25 mg/L |  |  |
| FeCl_3_·6H_2_O | 5 mg/L |  |  |
| soil extractablesolution | 40 mL |  |  |
| A5 Solution | 1 mL | H_3_BO_3_ | 286 mg/L |
|  |  | MnCl_2_·4H_2_O | 181 mg/L |
|  |  | ZnSO_4_·7H_2_O | 22 mg/L |
|  |  | CuSO_4_·5H_2_O | 7.9 mg/L |
|  |  | (NH_4_)_6_Mo_7_O_24_·4H_2_O | 3.9 mg/L |
| Fe-EDTA | 1 mL | Na-EDTA | 1 g |
|  |  | FeCl_3_·6H_2_O | 81 mg |
|  |  | HCl（0.1 mol/L） | 50 mL |
|  |  | distilled water | 50 mL |
| distilled water | 958 mL |  |  |

**Reagents and equipment.** The reagents and equipment involved in the test are shown in Table S3 and Table S4.

**Table S3** Reagents involved in the test

| Name | Purity | Manufacturer |
| --- | --- | --- |
| CLF | > 97 % | USA，Sigma-Aldrich |
| CBZ | > 99 % | USA，Sigma-Aldrich |
| DCF | > 99 % | USA，Sigma-Aldrich |
| CIP | > 98 % | USA，TCI |
| Acetonitrile | chromatographically pure | USA，Tedia |
| Phosphoric acid | chromatographically pure | USA，Tedia |
| Methanol | chromatographically pure | USA，Tedia |
| Acetic acid | chromatographically pure | USA，Tedia |
| Deionized water | — | Shanghai,Fudan Danhua |
| Cell lysate | — | Shanghai, Beyotime |
| PMSF | — | Shanghai，Beyotime |
| Hydrogen Peroxide Detection Kit | — | Shanghai，Beyotime |
| Total superoxide dismutase assay kit | — | Shanghai，Beyotime |
| BCA Protein Assay Kit | — | Shanghai，Beyotime |

**Table S4** Equipment involved in the test

| Equipment | Model | Manufacturers |
| --- | --- | --- |
| Liquid chromatography | Agilent 1200 | USA，Agilent |
| Light incubator | GZX-300BS-III | Shanghai，CIMO Medical Instrument |
| Malvern laser particle size analyzer | Mastersizer 3000 | UK，Malvern |
| Elemental analyzer | Vario EL III | Germany，ELEMENTAR |
| Fluorescence Spectrometer | F-4500 FL | Janpan，Hitachi |
| Microplate reader | Synergy™ 4 | USA，BioTek |
| Electronic balance | AL204-IC | Shanghai，Mettler-Toledo |
| UV spectrophotometer | UV-1101 | Shanghai，Techcomp |
| Ultra clean sterile workbench | BHC-1300ⅡA/B3 | Suzhou，Antai |

**Design of the test.** For quality control of the test process, the effect of potential influential factors, such as the effect of high-temperature sterilization, UV light and constant-temperature culture process on the loss of PPCPs, were studied before conducting the experiment.

**Effect of the SE medium on PPCP detection.** When detecting PPCPs, to exclude the influence of Ph or certain components in the SE medium on the peak position, peak area or overlap of the peaks with the PPCP culture medium, CLF, CIP and DCF were each dissolved in 100 mL of SE medium and 100 mL of deionized water to a concentration of 1, 5, 6.25, 10, 12.5, 25, 50, 100, and 120 mg/L; CBZ was dissolved in 100 mL of SE medium and 100 mL of deionized water to a concentration of 1, 5, 6.25, 10, 12.5, 25, 50, and 100 mg/L. After 30 min of setting, the samples were used for HPLC analysis.

**Effect of high-temperature sterilization on degradation of PPCPs.** CLF, CIP and DCF were each dissolved in 100 mL of SE medium to a concentration of 1, 5, 6.25, 10, 12.5, 25, 50, 100, and 120 mg/L. CBZ was dissolved in 100 mL of SE medium until it reached a concentration of 1, 5, 6.25, 10, 12.5, 25, 50, and 100 mg/L. HPLC analysis was conducted on the samples. The constituted PPCP solution was placed in a high-pressure steam sterilizer for sterilization at 120 °C for 30 min. After cooling, HPLC analysis was conducted on the samples, and the results were compared with the previous analysis results.

**Effect of ultraviolet light on degradation of PPCPs.** CLF, CIP and DCF were each dissolved in 100 mL of SE medium to a concentration of 1,5,6.25,10,12.5,25,50,100, and 120 mg/L. CBZ was dissolved in 100 mL of SE medium, reaching a concentration of 1, 5, 6.25, 10, 12.5, 25, 50, and 100 mg/L. Samples were taken for HPLC analysis. The constituted PPCP solution was placed on a clean bench with UV lamps for 30 min. HPLC analysis was conducted on the samples, and the results were compared with the previous analysis results.

**Effect of constant-temperature culture on degradation of PPCPs.** CLF, CIP and DCF were each dissolved in 100 mL of SE medium to a concentration of 1, 5, 6.25, 10, 12.5, 25, 50, 100, and 120 mg/L; CBZ was dissolved in 100 mL of SE medium to a concentration of 1, 5, 6.25, 10, 12.5, 25, 50, and 100 mg/L. HPLC analysis was conducted on the samples. After high-temperature sterilization, the solution was placed into the light incubator at a constant temperature for 8 d. HPLC analysis was conducted on the samples, and the results were compared with the previous analysis.

**Metabolomics analysis and data analysis of DCF on *C.pyrenoidosa* culture.**

**Detection method.** The 100 mg sample was ground in liquid nitrogen and transferred to a 10 mL centrifuge tube. Then, 1400μL 100% methanol (pre-cooled to -20 ℃) was added and the samples vortex oscillated for 30 s. A total of 60μL of 0.2 mg/mL ribose alcohol was used as the internal standard and the samples were vortex oscillated for 30 s, and then cleaned by ultrasonic cleaning machine for 15 min. Next, 750 μL of chloroform and 1400 μL of water were shaken, mixed, and centrifuged at 4000 rpm for 15 min. The supernatant was transferred to a new centrifuge tube and the sample dried with nitrogen. Then, 60 μL of 15 mg/mL methoxypyridine solution was added and vortexed for 30 s, and reacted overnight for 16 h. Finally, 60 μL of BSTFA reagent containing 1% trimethylchlorosilane was added and reacted at room temperature for 60 min. After these reactions, a sample with primary metabolite content was obtained. The [Agilent 7890-5975C Gas Chromatograph-Mass Spectrometer](http://www.google.com.hk/url?sa=t&rct=j&q=&esrc=s&source=web&cd=2&cad=rja&uact=8&ved=0ahUKEwiiz4mmtefSAhVCJ5QKHXcdAskQFgggMAE&url=http%3A%2F%2Fwww.gmu.edu%2Fdepts%2FSRIF%2Fequipment%2FGC-MS.html&usg=AFQjCNHUsOZHJsnW9bErw0jsLoBYuqVUyA) was used to detect the metabolite components of *C. pyrenoidosa*.

The chromatographic conditions were as follows:HP-5MS capillary column (5% phenyl methyl siloxane of 30 mx 250 μm id, 0.25 μm; Agilent;J & W Scientific, Folsom, CA); split injection, 1 μL injection, split ratio 20:1, inlet temperature of280 ℃; ion source temperature of 250 ℃; and interface temperature of 150 ℃. The programmed temperature was 80 °C and was maintained for 5 min. Then, the temperature was raised to 300℃ at 20 ℃/ min and maintained for 6 min. The total running time was 22 min. The carrier gas was helium. The carrier gas flow rate was 1 mL/min.

The MS conditions were as follows: electrospray ionization source, full scan mode, electron energy of 70 eV; and quadrupole scanning range of m/z 35 ~ 780.

**GC/MS data processing methods.** The original documents obtained by the Agilent 7890A/5975C GC/MS were pretreated with the GC-MS pretreatment software XCMS. The annotation databases used are the National Commercial Database of the National Institute of Standards and Technology (NIST) and the metabolomics database of the Wiley Registry. The metabolite alkane retention index is based on The Golm Metabolome Database (GMD) for further qualitative characterization.

**Statistical analyses.** The peak area of the normalized substance was chosen as variable X and the matrix was established. The data were introduced into SIMCA-P 13.0 (Umea, Sweden) statistical software for multivariate analysis. Principal component analysis (PCA) was used to analyze the clustering trend of the samples. A reliable statistical analysis Par model was established by partial least-squares discriminant analysis (PLS-DA). Biomarkers (variable VIP values> 1 biomarkers) were determined with the loading plot and the Variable Importance for the Projection (VIP) and p values of univariate statistical analysis lower than 0.05.

The product with significant metabolic differences will be used for further metabolic pathway analysis based on the KEGG database.

**Other analytical methods.** The absorbance measurement (UV-1101 UV/Visible Spectrophotometer, China) was used to quantify the concentration of *C. pyrenoidosa* at a wavelength of 680 nm. The OD680 value was converted to biomass concentration via appropriate calibration between OD680 and dry cell weight and the conversion factor was determined.^24^ The relationship of microalgae biomass concentration was shown as follows Eq.(1): (determined with the experiment result).

$\begin{matrix} Biomass & concentration & (g/L) \end{matrix}\begin{matrix} = & 0.2975\times OD_{680}-0.0216, \end{matrix}R^{2}=0.994$ (1)

Growth rate inhibition (ρ) was calculated according to the following Eq. (2) :

$$\mu=\frac{\ln N_{2}-\ln N_{0}}{t_{2}-t_{0}}$$

$\rho=100(\mu_{0}-\mu_{2})/\mu_{0}$ (2)

where N_2_ is the dry weight at time t_2_ and N_0_ is the dry weight at time t_0_ (day 0), μ_2_ is the growth rate at time t_2_ and μ0is the growth rate of control treatment.

To determine chlorophyll a concentration, a 5 mL culture was harvested by centrifugation at 4,500 rpm for 10 min. The cell pellets were resuspended in 5 mL of 95% methanol, incubated at 60°C for 5 min, and centrifuged again for 10 min. The absorbance of the supernatant at 665 and 652 nm was measured and the chlorophyll a concentration was calculated following Eq.(3):

$\begin{matrix} Chlorophyll & a & (mg/g)= \end{matrix}16.29\times A_{665}-8.54\times A_{652}$ (3)

Algal lipid extraction was based on the modified Soxhlet extractor method. PSD was performed by measuring the pattern of light scattered by particles in the sample (Beckman Coulter LS 230, USA). To extract antioxidant enzymes, 2 mL samples of each culture were centrifuged at 4,000 rpm for 10 min. The cell pellets were collected and separately seeded in 96-well plates. A 200 μl cell lysis buffer was added into each well, incubated for 5 min and centrifuged. Supernatants were collected and further assayed. The activity of superoxide dismutase (SOD) in the supernatant was determined using WST-1. Catalase activity (CAT) was determined by following the consumption of H_2_O_2_ at 240 nm during 1 min at 25 °C.

The HPLC analyses were performed on an Agilent 1200 (Agilent Technologies, USA) HPLC system using a Shimadzu C_18_ reversed-phase column (250×4.6 mm, particle size 5 μm). Absorbance was monitored at 230 nm with a column temperature 30 °C. Mobile phases were 0.1% phosphoric acid (phase A) and methanol: acetonitrile (1:1) with 0.1% acetic acid (phase B). The Phase A:Phase B ratio was 40%:60% (v:v). The flow rate was 1 mL/min. The injection volume was 20 μl.

**RESULTS AND DISCUSSIONS**

**The effect of SE medium on the detection of PPCPs.** The effect of SE medium on the detection of PPCPs is shown in Fig. S1. Fig. S1shows that the PPCP concentration in the SE medium has good correspondence with that in the deionized water, indicating that the SE medium did not affect detection of the PPCPs concentration. Therefore, the culture medium containing PPCPs could be used in the subsequent test procedure.


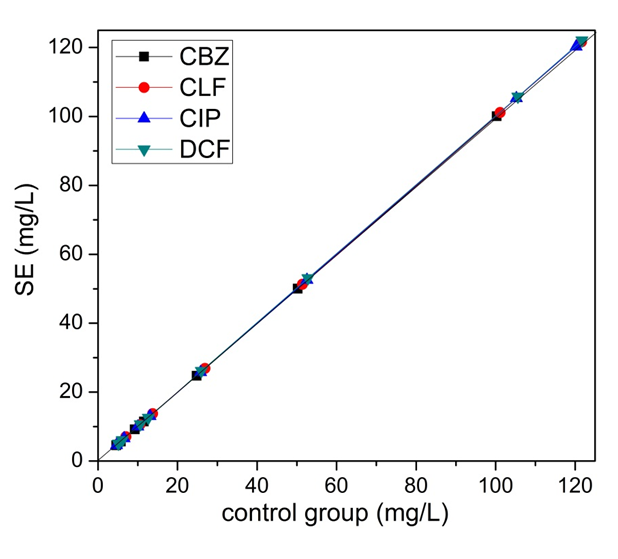


**Figure. S1** The effect of SE medium on detection concentration of PPCPs.

**The effect of** **high-temperature sterilization and UV light on degradation of PPCPs.** The effects of high-temperature sterilization and ultraviolet light on the degradation of PPCPs are shown in Fig. S2 and Fig. S3, respectively. As shown in Fig. S2, the high-temperature sterilization process had no effect on the concentration of PPCPs in the culture medium. Additionally, Fig. S3 illustrates that 30 min of UV light degrades a small portion of CLF and CBZ, but the degradation rate does not exceed 4%.


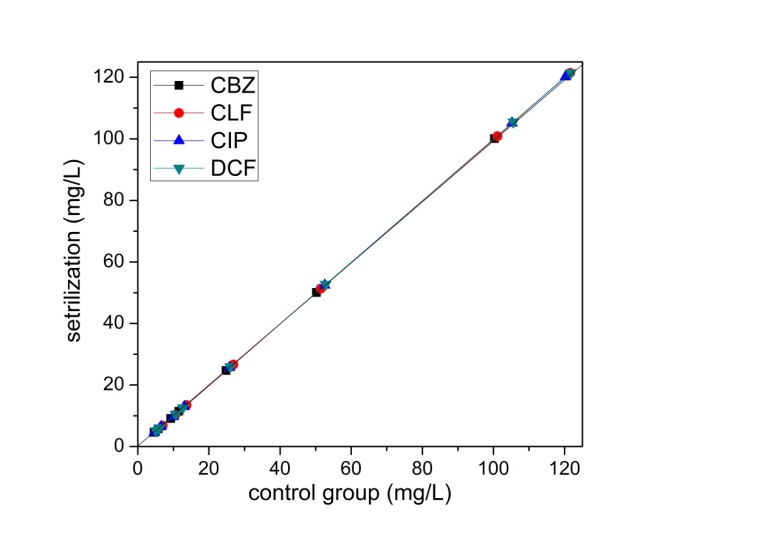


**Figure.S2** The effects of high temperature sterilization on degradation of PPCPs.


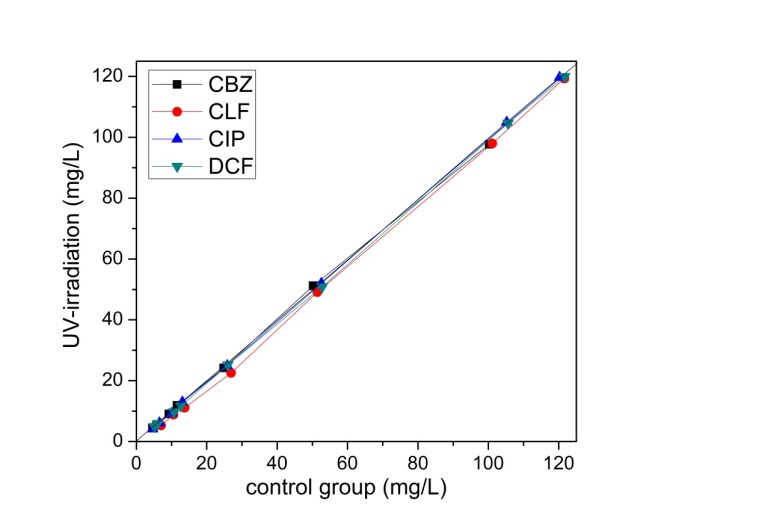


**Figure.S3** The effects of ultraviolet light on degradation of PPCPs.

**The effect of constant-temperature culture on degradation of PPCPs.** The effect of constant-temperature culture on degradation of PPCPs is shown in Fig. S4. According to Fig. S4, after 10 days of cultivation, the concentration of PPCPs in the culture medium decreased by approximately 0-4%. The SE culture medium and high-temperature sterilization process had no effect on the PPCP concentration, while UV light could degradethe PPCPs to less than 4% in the culture medium. In this study, UV light had the greatest impact on the degradation of CLF, and the degradation was 3.4%. Therefore, the degradation of PPCPs during the constant-temperature culture could be attributed mainly to the UV process that occurred before culture.


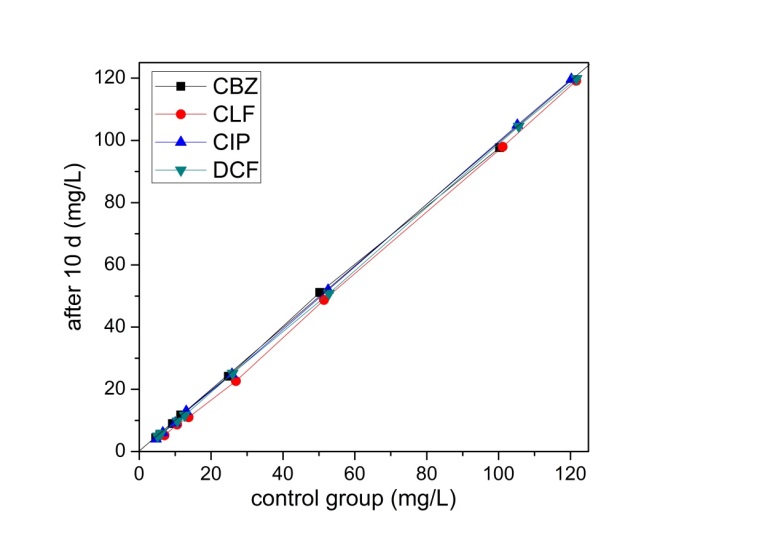


**Figure. S4** Effect of constant-temperature culture on degradation of PPCPs.

**Effect of DCF on the growth of *C. pyrenoidosa***


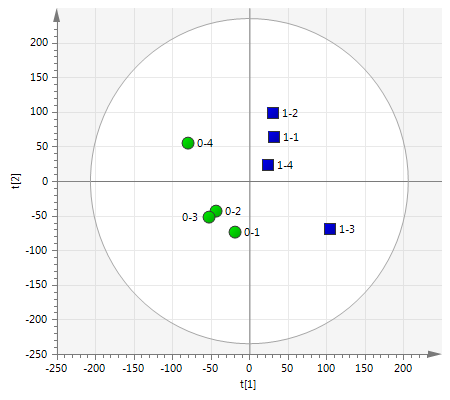


**Figure.S5** PLS-DA scatter plot of group 0 and group 1 (R^2^X = 0.864, R^2^Y = 0.957, Q^2^Y=0.887) (0-5contrast).


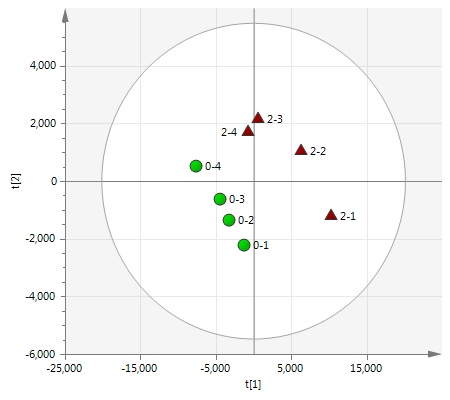


**Figure.S6** PLS-DA scatter plot of group 0 and group 2 (R^2^X = 0.871, R^2^Y = 0.979, Q^2^Y=0.945) (0-10contrast).


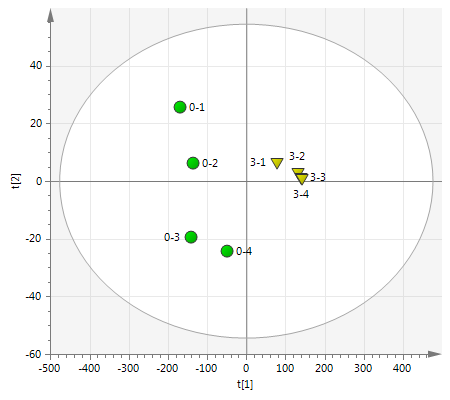


**Figure.S7** PLS-DA scatter plot of group 0 and group 3 (R^2^X = 0.972，R^2^Y = 0.959, Q^2^Y=0.892) (0-100contrast).


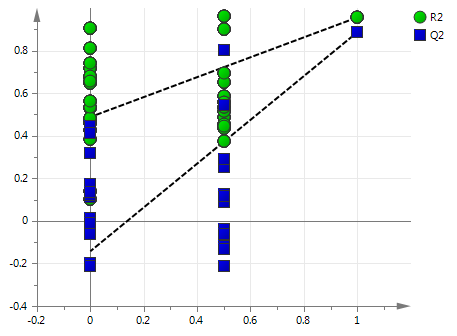


**Figure. S8** Overfitting of group 0 and group 1 with the PLS-DA model (R^2^-intercept=0.489，Q^2^-intercept=-0.142) (0-5 contrast).


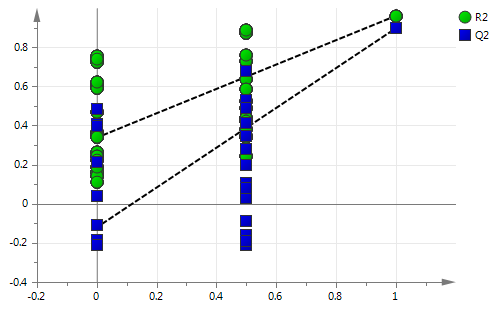


**Figure. S9** Overfitting of group 0 and group 2 with the PLS-DA model (R^2^-intercept=0.338，Q^2^-intercept=-0.12) (0-10 contrast).


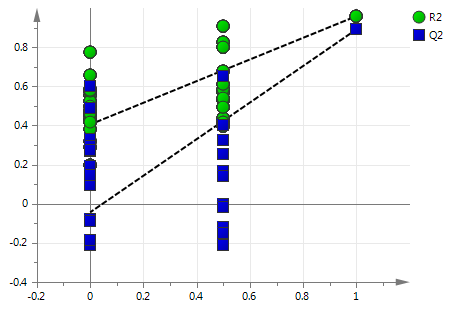


**Figure. S10** Overfitting of group 0 and group 3 with the PLS-DA model (R^2^-intercept=0.406，Q^2^-intercept=-0.043) (0-100 contrast).


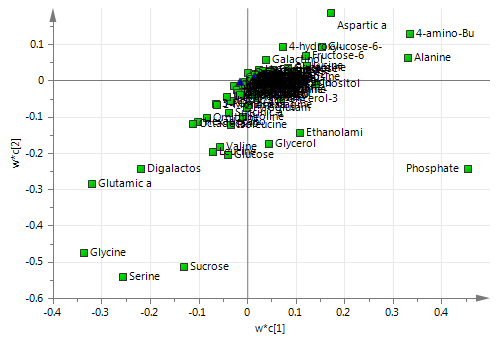


(0-5 mg/L contrast)


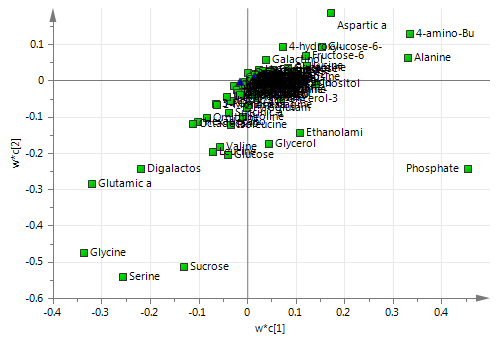


(0-10mg/L contrast)


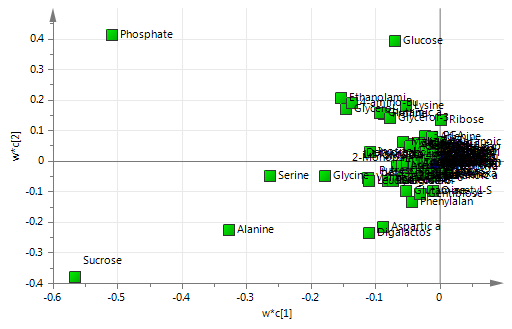


(0-100 mg/L contrast)

**Figure. S11** Loading plot


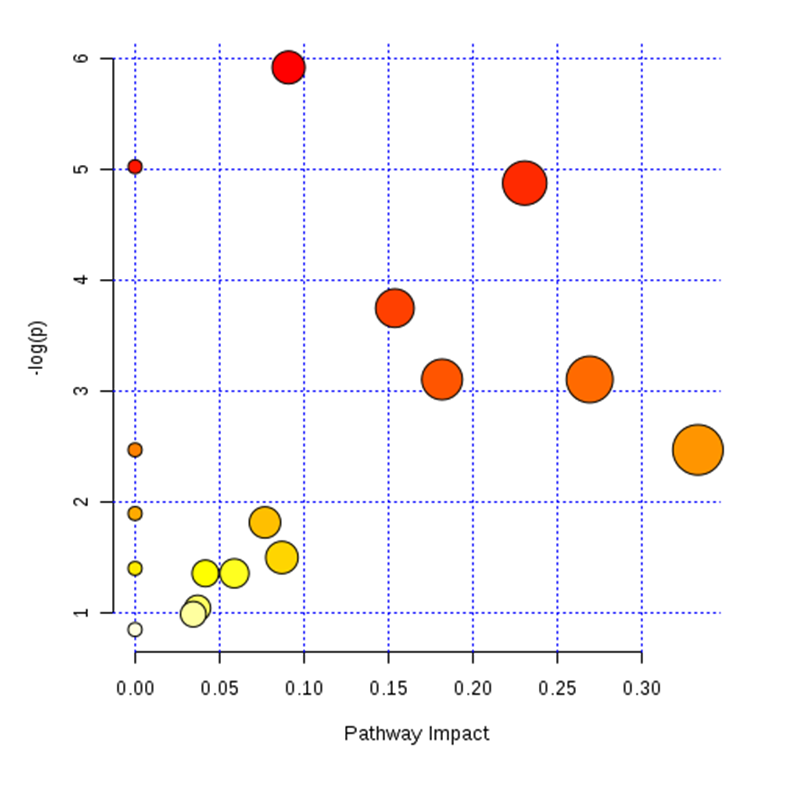


**1**

**2**

**3**

**4**

**5**

**6**

**7**

**8**

(5 mg/L group)


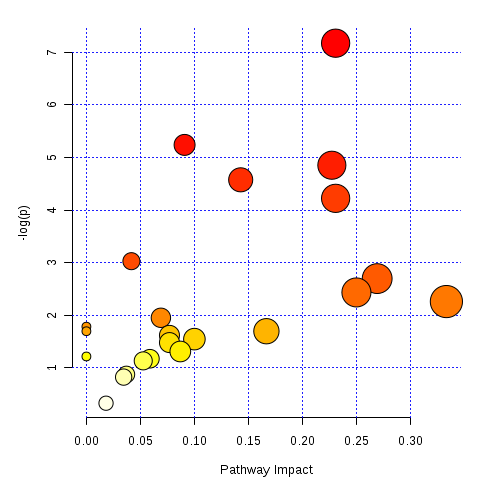


**1**

**2**

**3**

**4**

**5**

**6**

**7**

**8**

**9**

**10**

**11**

**12**

**13**

(10 mg/L group)


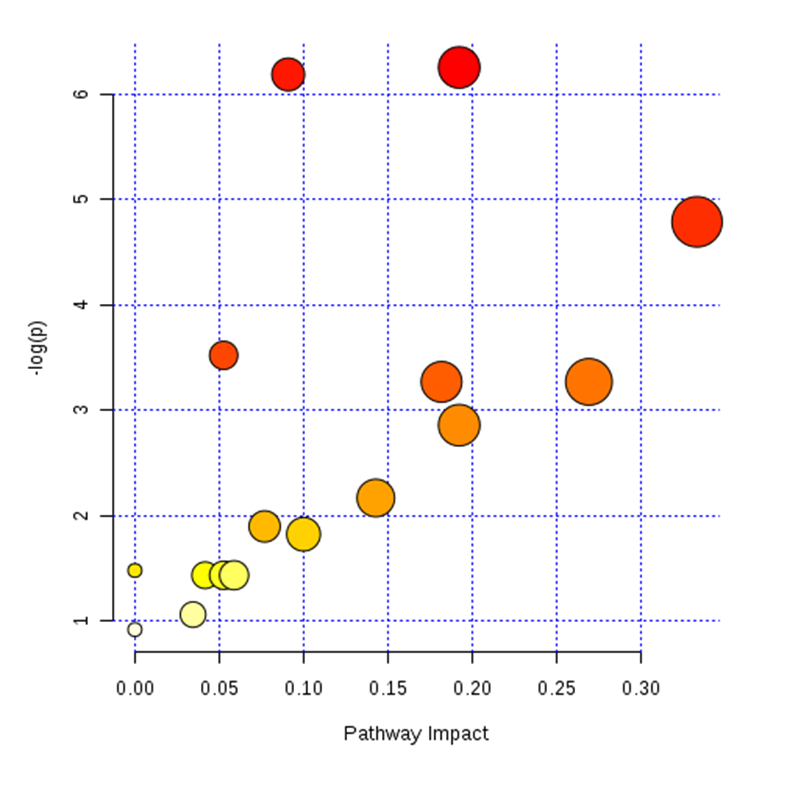


**1**

**2**

**3**

**4**

**5**

**6**

**7**

（100mg/L group）

**Figure. S12** Metabolic pathway topology analysis (the numbers in the figure correspond to the metabolic pathways in Fig. S8, S9, and S10, respectively).

**Table S5** Biomarkers between group 1 and the control group (0-5 contrast).

| Metabolite name | mz | RT | VIP | FC |
| --- | --- | --- | --- | --- |
| Phosphate | 299.1 | 9.48 | 4.4 | 1.361 |
| Glycine | 102.1 | 7.13 | 3.1 | 0.440 |
| Alanine | 116.1 | 6.88 | 2.8 | 1.354 |
| 4-amino-Butyric acid | 174.1 | 12.81 | 2.8 | 2.231 |
| Serine | 204.1 | 10.68 | 2.7 | 0.812 |
| Glutamic acid | 246.1 | 13.90 | 2.7 | 0.413 |
| Sucrose | 361.2 | 23.61 | 2.2 | 1.047 |
| Digalactosylglycerol | 204.1 | 27.47 | 1.9 | 0.535 |
| Aspartic acid | 232.1 | 12.73 | 1.5 | 1.668 |
| Glucose-6-phosphate | 299.1 | 21.05 | 1.3 | 2.048 |
| Ethanolamine | 174.1 | 9.34 | 1.3 | 1.340 |
| Inositol | 305.1 | 18.95 | 1.3 | 1.681 |
| Lysine | 174.1 | 17.15 | 1.0 | 1.978 |
| Fructose-6-phosphate | 315.1 | 20.94 | 1.0 | 2.188 |

Here, mz is the mass count of metabolites; RT is the metabolic retention time; FC is the ratio between the test group and the blank control group; FC> 1 indicates a higher metabolite content; and FC <1 indicates a lower metabolite content. These same definitions are used in Table S6-S7.

**Table S6** Biomarkers between group 2 and control group (0-10 contrast).

| Metabolite name | mz | RT | VIP | FC |
| --- | --- | --- | --- | --- |
| Phosphate | 299.1 | 9.48 | 4.7 | 1.79 |
| Alanine | 116.1 | 6.88 | 4.0 | 1.35 |
| 4-amino-Butyric acid | 174.1 | 12.81 | 3.4 | 5.80 |
| Glycine | 102.1 | 7.13 | 2.3 | 0.32 |
| Aspartic acid | 232.1 | 12.73 | 2.2 | 4.73 |
| Serine | 204.1 | 10.68 | 2.1 | 0.89 |
| Ethanolamine | 174.1 | 9.34 | 1.8 | 2.16 |
| Glycerol | 205.1 | 9.48 | 1.6 | 1.69 |
| Glycerol-3-phosphate | 357.1 | 15.59 | 1.5 | 3.58 |
| Digalactosylglycerol | 204.1 | 27.47 | 1.3 | 1.29 |
| Sucrose | 361.2 | 23.61 | 1.2 | 1.11 |
| Glutamic acid | 246.1 | 13.90 | 1.2 | 0.67 |
| Glucose | 319.1 | 17.04 | 1.1 | 2.03 |
| Lysine | 174.1 | 17.15 | 1.1 | 3.20 |
| Glucose-6-phosphate | 299.1 | 21.05 | 1.1 | 2.44 |
| Leucine | 158.1 | 9.42 | 1.0 | 0.79 |
| Fructose-6-phosphate | 315.1 | 20.94 | 1.0 | 3.06 |

**Table S7** Biomarkers between group 3 and control group (0-100 contrast).

| Metabolite name | mz | RT | VIP | FC |
| --- | --- | --- | --- | --- |
| Sucrose | 361.2 | 23.61 | 5.3 | 0.20 |
| Phosphate | 299.1 | 9.48 | 4.8 | 0.12 |
| Alanine | 116.1 | 6.88 | 3.1 | 0.09 |
| Serine | 204.1 | 10.68 | 2.4 | 0.06 |
| Glycine | 102.1 | 7.13 | 1.7 | 0.02 |
| Ethanolamine | 174.1 | 9.34 | 1.5 | 0.15 |
| Glycerol | 205.1 | 9.48 | 1.4 | 0.23 |
| 4-amino-Butyric acid | 174.1 | 12.81 | 1.3 | 0.07 |
| Digalactosylglycerol | 204.1 | 27.47 | 1.1 | 0.24 |
| Valine | 144.1 | 8.61 | 1.0 | 0.07 |
| Leucine | 158.1 | 9.42 | 1.0 | 0.07 |
| Glucose | 319.1 | 17.04 | 1.0 | 0.18 |

**Table S8** Analytical results of the metabolic pathway (5 mg/L group).

|  | Pathway Name | Total | Hits | p | -log(p) | Impact | Details |
| --- | --- | --- | --- | --- | --- | --- | --- |
| 3 | Methane metabolism | 30 | 3 | 0.0076324 | 4.8754 | 0.23076 | KEGG |
| 4 | Aminoacyl-tRNA biosynthesis | 45 | 3 | 0.023537 | 3.7492 | 0.15384 | KEGG |
| 5 | Glyoxylate and dicarboxylate metabolism | 24 | 2 | 0.044725 | 3.1072 | 0.18182 | KEGG |
| 6 | Glycine, serine and threonine metabolism | 24 | 2 | 0.044725 | 3.1072 | 0.26923 | KEGG |
| 8 | Lysine degradation | 6 | 1 | 0.084308 | 2.4733 | 0.33333 | KEGG |

**Table S9** Analytical results of the metabolic pathway (10 mg/L group).

|  | Pathway Name | Total | Hits | p | -log(p) | Impact | Details |
| --- | --- | --- | --- | --- | --- | --- | --- |
| 1 | Aminoacyl-tRNA biosynthesis | 45 | 5 | 7.6605E-4 | 7.1743 | 0.23076 | KEGG |
| 3 | Glyoxylate and dicarboxylate metabolism | 24 | 3 | 0.0078076 | 4.8527 | 0.22727 | KEGG |
| 5 | Methane metabolism | 30 | 3 | 0.014657 | 4.2228 | 0.23076 | KEGG |
| 7 | Glycine,serine and threonine metabolism | 24 | 2 | 0.067479 | 2.6959 | 0.26923 | KEGG |
| 8 | D-Glutamine and D-glutamate metabolism | 5 | 1 | 0.087766 | 2.4331 | 0.25 | KEGG |
| 9 | Lysine degradation | 6 | 1 | 0.10443 | 2.2592 | 0.33333 | KEGG |
| 13 | Valine, leucine and isoleucine degradation | 11 | 1 | 0.18359 | 1.6951 | 0.16667 | KEGG |
| 23 | Porphyrin and chlorophyll metabolism | 67 | 1 | 0.72203 | 0.32568 | 0.01818 | KEGG |

**Table S10** Analytical results of the metabolic pathway (100 mg/L group).

|  | Pathway Name | Total | Hits | p | -log(p) | Impact | Details |
| --- | --- | --- | --- | --- | --- | --- | --- |
| 1 | Aminoacyl-tRNA biosynthesis | 45 | 4 | 0.0019215 | 6.2547 | 0.1923 | KEGG |
| 3 | Valine, leucine and isoleucine degradation | 11 | 2 | 0.008313 | 4.7899 | 0.33334 | KEGG |
| 5 | Glyoxylate and dicarboxylate metabolism | 24 | 2 | 0.037937 | 3.2718 | 0.18182 | KEGG |
| 6 | Glycine, serine and threonine metabolism | 24 | 2 | 0.037937 | 3.2718 | 0.26923 | KEGG |
| 7 | Methane metabolism | 30 | 2 | 0.057231 | 2.8607 | 0.1923 | KEGG |
